# Value of SOFA, APACHE IV and SAPS II scoring systems in predicting short-term mortality in patients with acute myocarditis

**DOI:** 10.18632/oncotarget.18634

**Published:** 2017-06-27

**Authors:** Dating Sun, Hu Ding, Chunxia Zhao, Yuanyuan Li, Jing Wang, Jiangtao Yan, Dao Wen Wang

**Affiliations:** ^1^ Division of Cardiology, Department of Internal Medicine, Tongji Hospital, Tongji Medical College, Huazhong University of Science and Technology, Wuhan 430030, People's Republic of China

**Keywords:** myocarditis, mortality, scoring systems

## Abstract

Acute myocarditis is an uncommon and potentially life-threatening disease. Scoring systems are essential for predicting outcome and evaluating the therapy effect of adult patients with acute myocarditis. The aim of this study was to determine the value of the Sequential Organ Failure Assessment (SOFA), Acute Physiology and Chronic Health Evaluation IV (APACHE IV) and second Simplified Acute Physiology Score (SAPS II) scoring systems in predicting short-term mortality of these patients. We retrospectively analyzed data from 305 adult patients suffering from acute myocarditis between April 2005 and August 2016. The association between the value of admission SOFA, APACHE IV and SAPS II scores and risk of short-term mortality was determined. Multivariate Cox analysis showed that SOFA, APACHE IV and SAPS II scores were independent risk factors of death in patients with acute myocarditis. For each scoring system, Kaplan–Meier analysis showed that the cumulative short-term mortality was significantly higher in patients with higher admission scores compared with those with lower admission scores. For the prediction of short-term mortality in a patient with acute myocarditis, SAPS II had the highest accuracy followed by the APACHE IV and SOFA scores.

## INTRODUCTION

Myocarditis is an inflammatory disease of myocardium with a wide range of clinical presentations, from complete healing to severe congestive heart failure, leading to death or requiring a heart transplant [1]. Recently, some clinical markers or risk factors, including syncope [2], New York Heart Association (NYHA) Functional class [3], right ventricular dysfunction [4], acute kidney injury (AKI) [5] as well as elevated troponin I [6] and pulmonary artery pressure [7], presence of anti-heart autoantibodies [8] and prolonged QRS duration≥120 ms [9] have been shown to predict the elevated risks of cardiac death or heart transplantation in patients with acute myocarditis. In addition, extreme elevation of serum levels serum interleukin-10 and soluble Fas concentrations were associated with an increased risk of death in acute or fulminant myocarditis [10–12]. Moreover, immunohistologic signs of inflammation (CD3 and/or CD68) were also predictors of increased risk of death [3]. However, these markers were not commonly used for clinical test. Therefore, a risk stratification approach based on clinical findings providing information on the various myocarditis induced injuries could help us to identify patients with a potentially unfavorable prognosis.

SOFA score was a scoring system to determine the extent of a person's organ function or rate of failure according to 6 different scores, which represent the respiratory, cardiovascular (with adjustment for inotropic agents), hepatic, coagulation, renal, and neurological systems, respectively [13]. The first generation of APACHE score was developed in 1981 [14], which was revised and published in 1985 and 1991, respectively, and resulted in APACHE II and APACHE III [15–16]. APACHE IV score is the youngest APACHE score was introduced in 2006 [17] and used for estimating the risk of short-term mortality from actual clinical data in the first day after admissionas well as predicting the length of intensive care unit (ICU) stay [18]. SAPS II score was developed in 1993 [19] and is a frequently used severity-of-disease classification system. All these scores were risk stratification tools for estimating the risk of mortality in clinical applications. Up to date, however, there have been no reports concerning using these clinical scores to predict the survival of patients with acute myocarditis in the acute phase. The aim of this report was to assess the clinical utility of these scores to predict the risk of death from adult patients with acute myocarditis.

## RESULTS

### Clinical characteristics of patients

The baseline characteristics of 305 patients with clinically suspected acute myocarditis including demographic data, biochemical data and clinical parameters risk scores as well as details of echocardiography data, were shown in Table 1 and Supplementary Table 1, respectively. Among these patients, a total of 250 patients survived and 55 died during hospitalization, and therefore, the study subjects comprised survived and dead groups. There were statistically significant differences in nearly all the listed characteristics, except for percentage of sex, ESR, application of temporary pacing, left aortal atrium diameter, end-diastolic left ventricular diameter and intraventricular septum diameter (*p*>0.05) (Table 1 and Supplementary Table 1). The most importantly, as shown in Table 1, all the scores of SOFA (survival group 2 [IQR 0 to 5] versus death group 8 [IQR 7 to 9], *p*<0.001), APACHE IV (survival 11 [IQR 6 to 20] versus death 43 [IQR 34 to 53], *p*<0.001) and SAPS II (survival 13 [IQR 8 to 22] versus death 40 [IQR 29 to 51], *p*<0.001) were significantly higher in death group than those in survival group.

**Table 1:** Baseline clinical characteristics of patients with acute myocarditis

Clinical variables	Survival (n=250)	Death (n=55)	*p* Value
**Demographic data**			
Age (year)	35.02±0.97	42.67±2.23	0.001
Males, n (%)	145 (58)	33 (60)	0.454
**Biochemical data**			
CK (U/L)	8.00±0.13	9.48±0.32	<0.001
DH (U/L)	8.38±0.10	9.85±0.31	<0.001
ALT (U/L)	5.87±0.13	7.68±0.37	<0.001
AST (U/L)	6.35±0.12	8.71±0.38	<0.001
cTnI (ng/ml)	2.01±0.12	4.14±0.28	<0.001
NT-proBNP (pg/ml)	11.19±0.14	13.20±0.25	<0.001
CRP (mg/L)	3.92±0.11	5.69±0.19	<0.001
ESR (mm/H)	2.97±0.09	3.04±0.14	0.696
D-dimer (ug/mL)	2.38±0.36	8.14±1.23	<0.001
HCO3- (mmol/L)	22.47±0.27	16.63±0.72	<0.001
HCT (%)	38.90±0.35	36.49±0.93	0.018
WBC (*109/L)	9.69±0.32	13.44±1.09	0.002
PLT (*109/L)	198.6±5.9	160.4±14.4	0.008
Serum creatinine (mg/dL)	0.94±0.03	1.32±0.07	<0.001
BUN (mmol/L)	6.56±0.34	12.68±0.95	<0.001
eGFR (ml/min/1.73m2)	90.33±1.98	71.01±4.28	<0.001
UA (umol/L)	364.56±10.43	494.52±32.98	<0.001
**Clinical parameters**			
Admission SBP (mmHg)	111.28±1.36	89.42±3.43	<0.001
Admission DBP (mmHg)	69.59±0.95	55.89±2.53	<0.001
Hypotension, n (%)	44 (17.6)	28 (50.9)	<0.001
NYHA (III or IV), n (%)	69 (27.6)	43 (78.2)	<0.001
LVEF (%)	54.43±0.79	44.13±1.74	<0.001
Arrhythmia, n (%)	125 (50)	39 (70.9)	0.003
Admission duration (days)	11.26±0.45	5.60±0.96	<0.001
**Treatments, n (%)**			
Temporary pacing	19 (7.6)	3 (5.5)	0.414
Inotropic agents	65 (26.0)	47 (85.5)	<0.001
IABP support	16 (6.4)	12 (21.8)	0.001
**Risk scores, (IQR)**			
SOFA	2 (0-5)	8 (7-9)	<0.001
APACHE IV	11 (6-20)	43 (34-53)	<0.001
SAPS II	13 (8-22)	40 (29-51)	<0.001

n indicates number of individuals; CK, Creatine Kinase; LDH, Lactate Dehydrogenase; ALT, alanine aminotransferase; AST, Aspartate aminotransferase; cTnI, cardiac troponin I; CRP, C reactive protein; NT-proBNP, N-terminal B-type natriuretic peptide; HCT, red blood cell specific volume; ESR, erythrocyte sedimentation rate; WBC, white blood cell; PLT, platelet count; BUN, blood urea nitrogen; eGFR, Estimated glomerular filtration rate; UA, uric acid; SBP, systolic blood pressure; DBP, diastolic blood pressure; LVEF, left ventricular ejection fraction; IABP, intra-aortic balloon pump; IQR, interquartile range. Hypotension, MAP (mean arterial pressure) <70 mmHg; SOFA, Sequential Organ Failure Assessment; APACHE IV, Acute Physiology and Chronic Health Evaluation IV; SAPS II, second Simplifed Acute Physiology Score. Values are expressed as mean ± SE unless otherwise noted; test for differences between survived and dead groups.

### Correlation analysis

We performed correlation analysis between risk scores and parameters of cardiac function. As shown in Table 2, all the individual risk scores had positive correlations with NYHA functional class IV (r range from 0.516 to 0.534, all the *p*<0.001) and cTnI (r range from 0.389 to 0.429, all the *p*<0.001) and had negative correlations with the admission LVEF (r range from −0.418 to −0.349, all the *p*<0.001).

**Table 2:** Partial correlations between clinical or biochemical parameters with SOFA, APACHE IV and SAPS II scores

	SOFA		APACHE IV		SAPS II	
	r	*p*	r	*p*	r	*p*
NYHA functional class IV	0.534	<0.001	0.554	<0.001	0.516	<0.001
cTnI (ng/ml)	0.429	<0.001	0.409	<0.001	0.389	<0.001
LVEF (%)	−0.396	<0.001	−0.418	<0.001	−0.349	<0.001

SOFA, Sequential Organ Failure Assessment score; APACHE IV, Acute Physiology and Chronic Health Evaluation IV score; SAPS II, second Simplifed Acute Physiology Score; cTnI, cardiac troponin I; LVEF, left ventricular ejection fraction; r, correlation coefficient; *p*, *p* value.

### Univariate and multivariate cox analysis for in hospital mortality

The univariate analysis of predictors of hospital short-term mortality showed that a total of 28 variables were associated with hospital short-term mortality (Table 3). Notably, admission SOFA, APACHE IV and SAPS II scores were associated with hospital short-term mortality all as a continuous variable (for SOFA score, HR=1.903, 95% CI 1.63–2.22, *p*<0.001; for APACHE IV score, HR=1.145, 95% CI 1.11–1.18, *p*<0.001; for SAPS II score, HR=1.219, 95% CI 1.16–1.28, *p*<0.001), respectively.

**Table 3:** Univariate analysis for hospital short-term mortality of patients with acute myocarditis

Clinical variables	HRs	95% CI	*p* Value
Age (year)	1.031	1.01 to 1.05	0.001
Males, n (%)	0.921	0.51 to 1.67	0.785
CK (U/L)	1.415	1.21 to 1.66	<0.001
LDH (U/L)	1.721	1.40 to 2.12	<0.001
ALT (U/L)	1.373	1.21 to 1.55	<0.001
AST (U/L)	1.500	1.32 to 1.71	<0.001
cTnI (ng/ml)	1.613	1.38 to 1.88	<0.001
NT-proBNP (pg/ml)	1.795	1.47 to 2.20	<0.001
CRP (mg/L)	1.851	1.51 to 2.27	<0.001
ESR (mm/H)	1.039	0.83 to 1.30	0.739
D-dimer (ug/mL)	1.126	1.07 to 1.19	<0.001
HCO_3_^−^ (mmol/L)	1.279	1.19 to 1.38	<0.001
HCT (%)	1.068	1.02 to 1.12	0.009
WBC (*10^9^/L)	1.103	1.05 to 1.16	<0.001
PLT (*10^9^/L)	1.005	1.00 to 1.01	0.008
Serum creatinine (mg/dL)	3.994	2.20 to 7.26	<0.001
BUN (mmol/L)	1.144	1.09 to 1.20	<0.001
eGFR (ml/min/1.73m^2^)	1.021	1.01 to 1.03	<0.001
UA (umol/L)	1.003	1.00 to 1.00	<0.001
Admission SBP (mmHg)	1.044	1.03 to 1.06	<0.001
Admission DBP (mmHg)	1.054	1.03 to 1.08	<0.001
Hypotension, n (%)	4.855	2.61 to 9.03	<0.001
NYHA (III or IV), n (%)	9.400	4.68 to 18.88	<0.001
LVEF (%)	1.059	1.04 to 1.08	<0.001
Arrhythmia, n (%)	2.437	1.30 to 4.59	0.006
Temporary pacing, n (%)	0.701	0.20 to 2.46	0.579
Inotropic agents, n (%)	0.060	0.03 to 0.13	<0.001
IABP support, n (%)	0.245	0.11 to 0.55	0.001
SOFA score	1.903	1.63 to 2.22	<0.001
APACHE IV score	1.145	1.11 to 1.18	<0.001
SAPS II score	1.219	1.16 to 1.28	<0.001

CK, Creatine Kinase; LDH, Lactate Dehydrogenase; cTnI, cardiac troponin I; NT-proBNP, N-terminal B-type natriuretic peptide; CRP, C reactive protein; ESR, erythrocyte sedimentation rate; HCT, Red blood cell specific volume; WBC, white blood cell; PLT, platelet count; BUN, blood urea nitrogen; eGFR, Estimated glomerular filtration rate; UA, uric acid; SBP, systolic blood pressure; DBP, diastolic blood pressure; Hypotension, MAP (mean arterial pressure) <70 mmHg; LVEF, left ventricular ejection fraction; IABP, intra-aortic balloon pump; SOFA, Sequential Organ Failure Assessment; APACHE IV, Acute Physiology and Chronic Health Evaluation IV; SAPS II, second Simplifed Acute Physiology Score.

Multivariable-adjusted HRs for short-term mortality according to 1-SD, the upper half and tertiles of SOFA, APACHE IV and SAPS II scores were presented in Table 4. Every admission risk score was an independent predictor of short-term mortality when considered as a continuous variable and as a categorical variable (the upper half) and stratified by tertiles (the highest tertile) after adjustment for other risk factors (all the *p*<0.001).

**Table 4:** Multivariate Cox analysis for hospital short-term mortality of patients with acute myocarditis

Clinical variables	HRs (95% CI)	*p* Value
**SOFA**		
Model 1		
SOFA score continuous 1-SD	1.53 (1.33 to 1.75)	<0.001
Model 2		
Lower Half (<3)	reference	
Upper Half (≥3)	6.80 (1.85 to 25.01)	0.004
Model 3		
Tertile 1 (<2)	reference	
Tertile 2 (2∼4)	3.40 (0.67 to 17.20)	0.140
Tertile 3 (>4)	12.16 (2.36 to 62.57)	0.003
**APACHE IV**		
Model 1		
APACHE IV score continuous 1-SD	1.09 (1.06 to 1.13)	<0.001
Model 2		
Lower Half (<17)	reference	
Upper Half (≥17)	7.95 (2.25 to 28.16)	0.001
Model 3		
Tertile 1 (<11)	reference	
Tertile 2 (11∼23)	4.23 (0.48 to 37.01)	0.192
Tertile 3 (>23)	33.86 (4.08 to 280.82)	0.001
**SAPS II**		
Model 1		
SAPS II score continuous 1-SD	1.14 (1.09 to 1.18)	<0.001
Model 2		
Lower Half (<14)	reference	
Upper Half (≥14)	7.15 (1.90 to 26.87)	0.004
Model 3		
Tertile 1 (<11)	reference	
Tertile 2 (11∼17)	3.90 (0.43 to 35.19)	0.226
Tertile 3 (>17)	26.45 (2.82 to 248.10)	0.004

HRs: hazard ratios; CI: confidence interval; SD: standard deviation; SOFA, Sequential Organ Failure Assessment score; APACHE IV, Acute Physiology and Chronic Health Evaluation IV score; SAPS II, second Simplifed Acute Physiology Score. Variables included in the multiple Cox analysis were age, CK, Creatine Kinase, LDH, Lactate Dehydrogenase, cTnI, cardiac troponin I, ALT, alanine aminotransferase, AST, Aspartate aminotransferase, NT-proBNP, N-terminal B-type natriuretic peptide, CRP, C reactive protein, D- dimer, HCO_3_^−^, HCT, Red blood cell specific volume, WBC, white blood cell, PLT, platelet count, Serum creatinine, BUN, blood urea nitrogen, eGFR, Estimated glomerular filtration rate, UA, uric acid, SBP, systolic blood pressure, DBP, diastolic blood pressure, NYHA (III or IV), LVEF, left ventricular ejection fraction and Arrhythmia.

**Figure 1:**
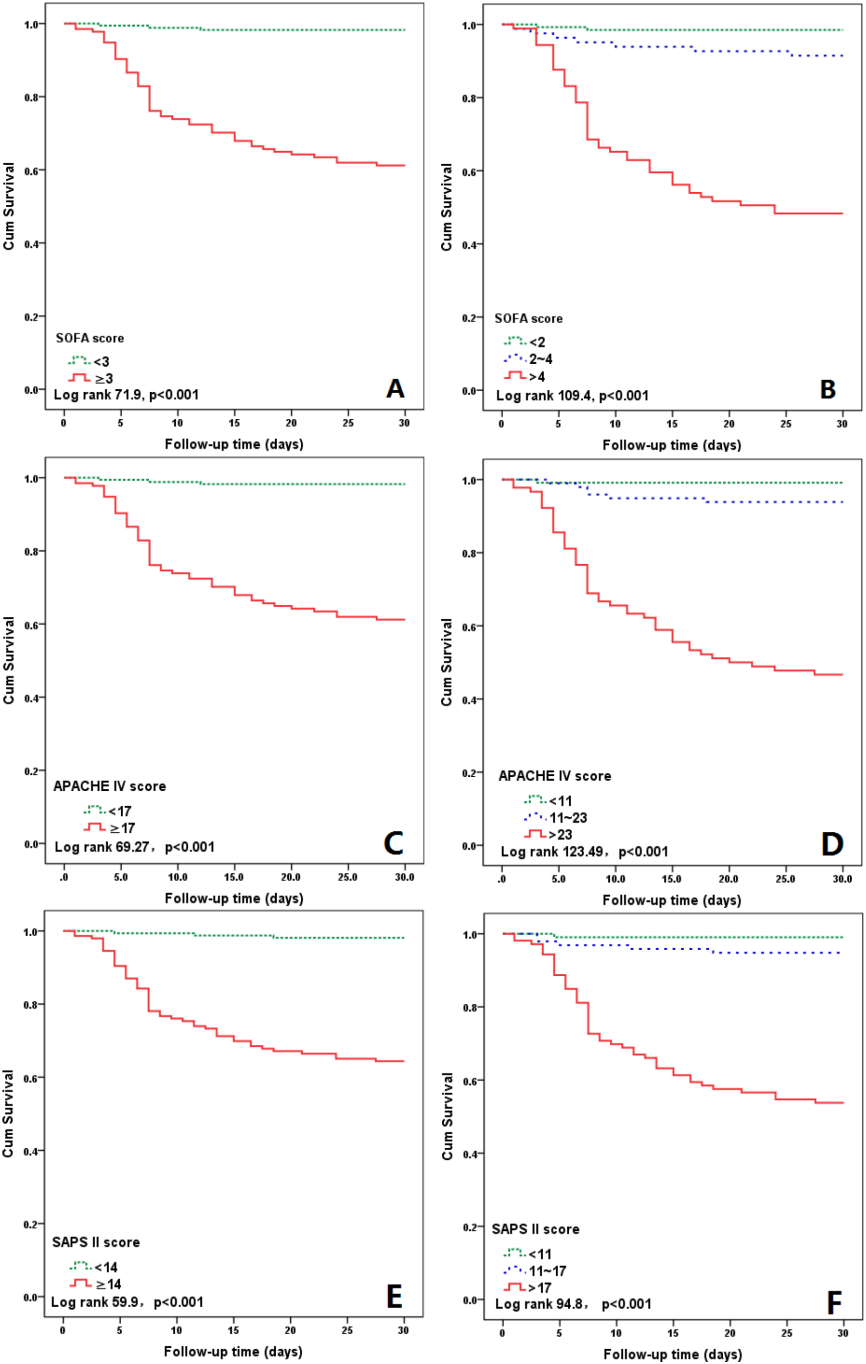
Kaplan–Meier curves for 30 days’ survival according to the medians of SOFA score (3), APACHE IV score (17) and SPAS II score (14) **(A, C, E)** and the tertiles of the three scores **(B, D, F)**.

As shown in Figures 1A–1F, Kaplan–Meier analysis showed that the cumulative short-term mortality was significantly higher in patients with admission upper half risk scores (SOFA, APACHE IV and SAPS II) compared with those with admission lower half risk scores (all the Log rank *p*<0.001). When stratified by risk scores tertiles, the short-term mortality significantly increased with all the risk scores increase (all the Log rank *p*<0.001).

### Sensitivity and specificity of SOFA, APACHE IV and SAPS II scores in predicting hospital mortality

**Figure 2:**
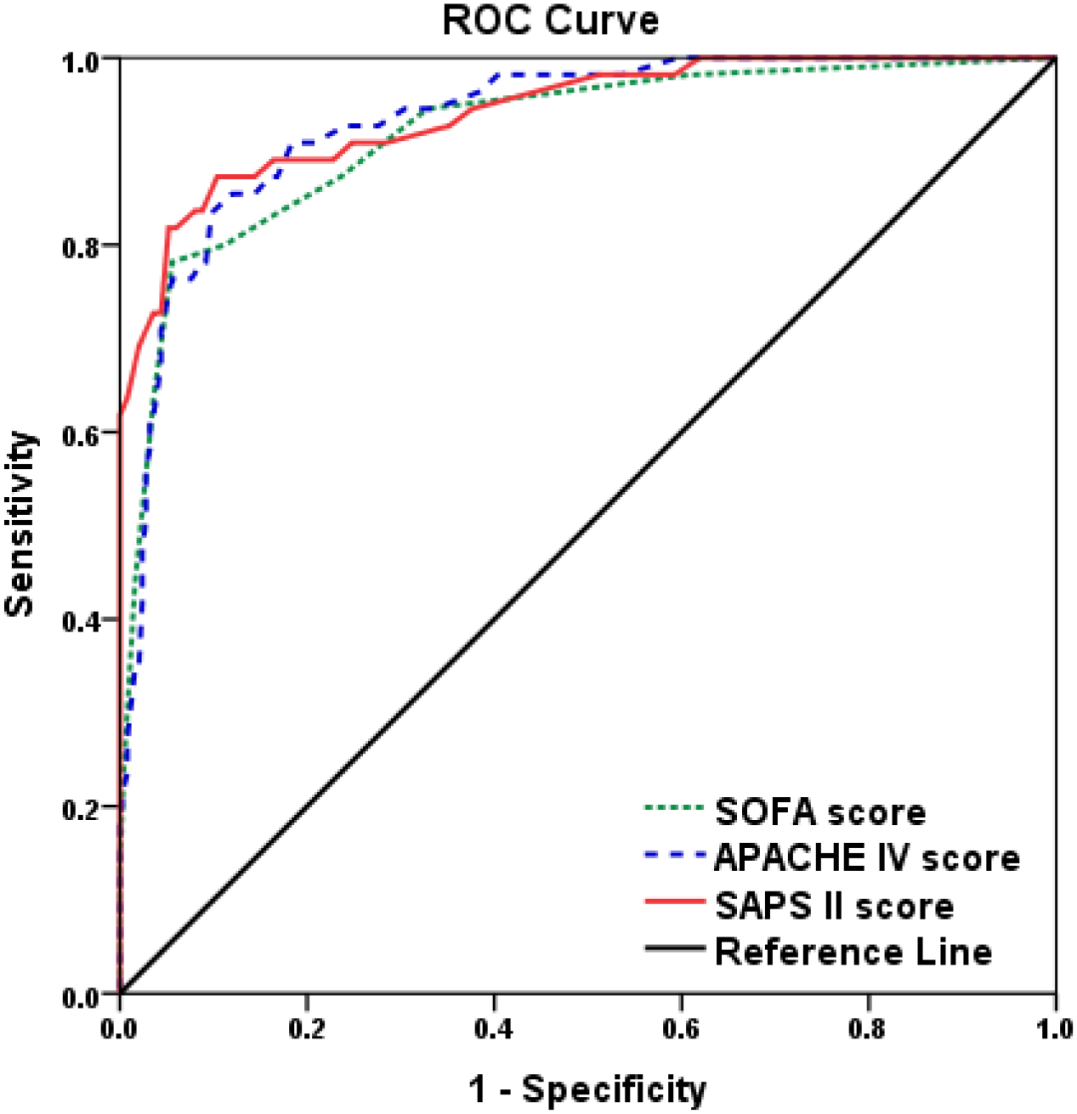
Diagnostic value of SOFA, APACHE IV and SAPS II scores for short-term mortality of patients with acute myocarditis

We performed receiver operating characteristic (ROC) analysis to determine the cut-off value of these risk score in evaluating hospital mortality. The cut-off values were 6.5 points, 28.5 points and 23.5 points for SOFA, APACHE IV and SAPS II scores with their higher sensitivity and specificity (Figure 2 and Table 5), respectively. The area under the curve (AUC) was 0.920, 0.934 and 0.942 for SOFA, APACHE IV and SAPS II scores, respectively. Meanwhile, when the sensitivity and specificity of SAPS II score reach to 100%, the higher specificity (38.0%) and sensitivity (61.8%) was obtained, compared with SOFA or APACHE IV score (Table 6).

**Table 5:** Diagnostic value of SOFA, APACHE IV and SAPSII scores for hospital short-term mortality of patients with acute myocarditis

	AUC	Cut-off value	Sensitivity (%)	Specificity (%)
SOFA	0.920	6.5	78.2	94.4
APACHE IV	0.934	28.5	83.6	90.0
SAPS II	0.942	23.5	87.3	89.6

SOFA, Sequential Organ Failure Assessment score; APACHE IV, Acute Physiology and Chronic Health Evaluation IV score; SAPS II, second Simplifed Acute Physiology Score; AUC, area under the curve.

**Table 6:** The sensitivity and specificity in predicting hospital short-term mortality of patients with acute myocarditis with different SOFA, APACHE IV and SAPS II scores cut-off values

	Cut-off value	Sensitivity (%)	Specificity (%)
SOFA	NA	100	NA
	10.5	12.7	100
APACHE IV	9.5	100	40.4
	56	18.2	100
SAPS II	9.5	100	38.0
	36.5	61.8	100

SOFA, Sequential Organ Failure Assessment score; APACHE IV, Acute Physiology and Chronic Health Evaluation IV score; SAPS II, second Simplifed Acute Physiology Score. NA, not available.

## DISCUSSION

The major novel findings of our study were that nonsurviving patients with acute myocarditis showed substantially higher risk scores, including SOFA, APACHE IV and SAPS II scores, than survivors. All these clinical scores could be used to predict short-term mortality in acute myocarditis patients, thus providing an additional level of risk stratification in patients with acute myocarditis.

SOFA, APACHE IV and SAPS II scores were designed to describe multiple organ function. Previous investigations have identified a clear relationship between organ dysfunction and mortality [20–21]. The main interest of the present study was to determine whether these scoring systems at the time of admission were able to predict hospital death in patients with acute myocarditis. In our patients, an elevated SOFA, APACHE IV and SAPS II scores were strong associated with cardiac dysfunction and circulatory failure, including clinical parameters of NYHA functional class IV and LVEF. Meanwhile, when SOFA, APACHE IV and SAPS II scores were all above the cut-off levels, the risk of death was substantially increased and death may occur. Our results might suggest that the SAPS II scoring systems was the best for predicting mortality in patients with acute myocarditis, although this difference among these scoring systems did not reach to statistical significance (data not shown). In previous studies, SAPS II scoring systems also exhibited the highest accuracy to predict mortality among critically ill patients in emergency department [22]. Thus, our findings may guide clinicians implement appropriate treatment strategies in emergent setting for acute myocarditis. In addition, early recognition of myocarditis with unfavorable outcome and early mechanical support might decrease the associated mortality rate.

Our data show that the shot-term (30-days) mortality rate is 18.0% (55 death of 305 patients) and it is really high compared with previous other studies with the sudden cardiac death rate of myocarditis in young adults from 8.6% to 12% [2, 23–24]. The major reasons of the high mortality rate may be explained by several possibilities: (1) Because of expensive medical costs, mechanical support, such as extracorporeal membrane oxygenation (ECMO) and IABP, were not available for all the patients. Thus, many critical patients couldn't get effective treatment in maintaining the stability of hemodynamic. (2) Due to many circulatory failure patients (i.e. hemodynamically not stable patients) were send to our center from local hospitals. The severity of cardiac dysfunction or circulatory failure might lead to differences in prognosis in different patient populations. (3) Given that shot-term mortality rate of present study based on our single center, this clinical outcome should be interpreted with caution.

There were still some limitations in our study. First, acute myocarditis was a clinical diagnosis in most cases, and rare patients (3 of 305 patients, less than 1%) received routine endomyocardial biopsy to confirm the diagnosis. In addition, although cardiac magnetic resonance (CMR) is a valuable tool in diagnosing acute myocarditis [25], CMR imaging was seldom used (5 of 305 patients) in the present study. However, combining the clinical features of viral myocarditis and substantial improvement in the left ventricular function supports a clinical diagnosis of active myocarditis. Second, this study was a retrospective, single-center study, which had a relatively small sample size, reducing the power of our stratification analyses. We consider that further multi-center studies employing a larger population are mandatory for confirming the correlation among scoring systems and the prognosis of acute myocarditis. Finally, the utility of these scoring systems for the identification of patients with acute myocarditis at high-risk of long-term mortality was not determinate in present study. Thus, additional research is needed to understand the role of admission scoring systems in the risk stratification of acute myocarditis.

In conclusion, a higher of SOFA, APACHE IV and SAPS II scores at the time of admission for patients with acute myocarditis may be an important consideration for determining clinical treatment. Of these scores, SAPS II score was more accurate and is easy to calculatein predicting the individual patient's short-time prognosis without additional medical costs. Thus, use of the SAPS II in cardiac intensive care units (CCU) for patients with acute myocarditis is recommended. However, our findings still need larger sample and prospective clinical studies to be further verified in order to contribute to prognostic assessment in patients with acute myocarditis.

## MATERIALS AND METHODS

### Study subjects

This study was approved by the institutional review board of Tongji Hospital, Wuhan, China. Owing to no breach of privacy and interference with clinical decisions related to patient care, informed consent was waived. All the subsequent experiments were conducted according to the principles expressed in the Declaration of Helsinki. Between April 2005 and August 2016, a total of 312 patients with a diagnosis of acute myocarditis at the Tongji Hospital were enrolled in a retrospective medical records review. All the acute myocarditis patients were diagnosed clinically according to the clinical features of acute heart failure following recent flu-like symptoms, or according to the Dallas criteria [26]. Patients diagnosed with a vague history of acute viral illness, coronary heart disease, cardiomyopathy, and genetic disease, as well as myocarditis secondary to sepsis, toxins, Kawasaki disease or arrhythmias were excluded from the study. Of these 312 patients, one patient under the age of 18 years were excluded, four were excluded due to incomplete data, and two were excluded because coronary angiography revealed substantial obstructive coronary artery disease. Finally, a total of 305 patients with acute myocarditis were evaluated. Among these patients, 250 patients survived and 55 died in a short term (it was defined short-term mortality when patient died in 30 days). Thus, the study subjects were divided into two groups: survival and death.

### Clinical characteristics of patients

All baseline vital signs were recorded at admission (systolic or diastolic blood pressure, heart rates, temperature), it was defined hypotension when MAP (mean arterial pressure)<70 mmHg. All patients underwent standard trans-thoracic echocardiography using a Vivid 7 ultrasound machine (GE Medical Systems, Connecticut, USA) at the time of admission. Based on echocardiographic data, we investigated the left ventricular ejection fraction, left atrium diameter, left ventricular end-systolic dimensions, thicknesses of the left ventricular post wall and the maximal interventricular septum. In addition, all the obtained Electrocardiography (ECG) results for the patient were collected, and the rhythm, heart rate, PR interval, QRS duration, QTc interval, and ST segment/T-wave changes were analyzed. All intervals were determined using commercial ECG analysis software (12-Lead Algorithm, GE Medical Systems, Connecticut, USA).

Venous blood was drawn from acute myocarditis patients in a fasting state within 24 hours of admission. Biochemical and hematological variables were analyzed on the Modular DP (Roche Diagnostics) and LH750 (Beckman Coulter), respectively. Levels of serum cardiac troponin I (cTnI) were measured using the third-generation Enzyme cTnI assay (Roche, Indianapolis, Indiana). Other variables, including leucocytes, erythrocytes, haemoglobin, platelet count, red blood cell specific volume (HCT), creatinine, N-terminal B-type natriuretic peptide (NT-proBNP), alanine aminotransferase (ALT), Aspartate aminotransferase (AST), creatine kinase (CK), lactate dehydrogenase (LDH), C reactive protein (CRP), erythrocyte sedimentation rate (ESR), blood urea nitrogen (BUN), uric acid (UA) and HCO_3_^−^ were determined by standard quantitative assay techniques in the hospital Clinical Laboratory Centers according to the manufacturers’ instructions. Estimated glomerular filtration rate (eGFR) was computed by using the abbreviated Modification of Diet in Renal Disease (MDRD) equation [27]. Potential virus infection were detect from serum using molecular techniques or direct immunofluorescence assay. The details etiology of patients with acute myocarditis can be found in Supplementary Table 2.

In cases where the blood pressure was low according to age, we initially administered dopamine; in the absence of a response, we added aramine or epinephrine. If cardiogenic shock persisted despite the administration of full medical support, ventricular ejection fraction (IABP) was considered early for patients with acute myocarditis when maximal pharmacological therapy failed.

SOFA and SAPS II scores were determined on the first day of treatment using an online calculator (http://clincalc.com), while APACHE IV score was calculated on the website (http://www.mecriticalcare.net/icu_scores/apacheIV.php) as previously describe [28].

### Statistical analysis

Statistical analysis was performed with SPSS 22.0 (SPSS Inc., Chicago, Illinois, USA) for Windows (Microsoft Corp, Redmond, Washington, USA). The distributions of quantitative variables were tested for normality by use of a 1-sample Kolmogorov-Smirnov test. Quantitative variables were compared by means of t test and analysis of variance for normally distributed data, and nonparametric Mann–Whitney U test for abnormally distributed data (SOFA, APACHE IV and SAPS II scores). Qualitative variables were compared by χ2 test or Fisher's exact test. Because observed cTnI, NT-proBNP, CRP, ESR, CK, LDH, ALT and AST were not normally distributed, we performed the natural-log transformation before statistical analysis. Spearman's rank correlation was used for assessing Correlations. Univariate and multivariate Cox proportional hazards analyses were used to analyze the predictive role of all the scoring systems for short-term mortality with hazard ratios (HRs) and 95% confidence intervals (CIs). Three separate multivariable Cox models were constructed with these scores entered as a continuous variable or as a categorical variable (the upper half) or stratified by tertiles (the highest tertile). Other variables selected for testing in the multivariate analysis were those with a *p*<0.05 in the univariate models. Kaplan–Meier analysis was performed to determined value of admission of SOFA, APACHE IV and SAPS II scoring systems in the prediction of short-term mortality and compared using the Log rank test. An optimal cut-off value for the continuous these scoring systems were calculated by applying a receiver operating curve analysis to discriminate between the survival and death groups. A *p* Value<0.05 was considered statistically significant. All probability values were 2-sided.

## SUPPLEMENTARY MATERIALS TABLES



## Abbreviations

SOFA: Sequential Organ Failure Assessment
APACHE IV: Acute Physiology and Chronic Health Evaluation IV
SAPS II: second Simplified Acute Physiology Score
CI: Confidence Interval
HR: Hazard Ratio
IQR: interquartile range
ROC: receiver operating characteristic
AUC: Area Under Curve
NYHA: New York Heart Association
SBP: systolic blood pressure
DBP: diastolic blood pressure
LVEF: Left Ventricular Ejection Fraction
