# Supplementary material for: Value of SOFA, APACHE IV and SAPS II scoring systems in predicting short-term mortality in patients with acute myocarditis
Source: Oncotarget. 2017 Jun 27;8(38):63073–83. doi: 10.18632/oncotarget.18634 (PMC5609904; doi:10.18632/oncotarget.18634)
Supplement: Supplementary file 1 [file oncotarget-08-63073-s001.pdf]

Value of SOFA, APACHE IV and SAPS II scoring systems in predicting short-term mortality in patients with acute myocarditis

SUPPLEMENTARY TABLES

Supplementary Table 1: Echocardiographic data of patients with acute myocarditis

Clinical variables	Survival (n=250)	Death (n=55)	*p* Value
echocardiographic parameters			
LVEF (%)	54.43±0.79	44.13±1.74	<0.001
LA dimension (mm)	33.18±0.57	35.73±1.16	0.213
LVEDd (mm)	48.71±0.58	49.04±1.12	0.059
IVS (mm)	10.33±0.46	10.23±0.48	0.403
Ventricular wall motion abnormalities, n (%)	96 (38.4%)	40 (72.7%)	<0.001

LVEF, left ventricular ejection fraction; LA, left aortal atrium; LVEDd, end-diastolic left ventricular diameter; IVS, intraventricular septum diameter. Values are expressed as mean ± SE; test for differences between survived and dead groups.

Supplementary Table 2: Etiology of patients with acute myocarditis

Etiologies	Survival	Death	*p* Value
Virus[a]			
Coxsackie virus,n	16	5	0.422
Epstein-Barr virus,n	13	3	0.574
Cytomegalovirus,n	9	3	0.468
Rubella virus,n	6	2	0.522
HSV1,n	3	1	0.610
Influenza A virus,n	4	1	0.729
Influenza B virus,n	3	0	0.496
Parvovirus B19,n	2	0	0.629
Echovirus,n	1	0	0.795
HPIVs,n	1	0	0.795
Bacterial			
Mycoplasma pneumonia,n	8	2	0.565
Toxins			
Cocaine,n	0	1	0.180
Paint,n	1	0	0.820

[a]Data on determinations of virus infection were available for 140 survival and 36 death participants; n indicates number of individuals; HSV1, herpes simplex virus type 1; HPIVs, human parainfluenza virus. Values are expressed as number of participants; test for differences between survived and dead groups.
